# The Physiology and Pathology of the Mouth

**Published:** 1860-04

**Authors:** 


### DENTAL REGISTER OF THE WEST.

VOL. XIII.] APRIL, 1860. [No. 10.

### Original Essays and Communications.

### THE PHYSIOLOGY AND PATHOLOGY OF THE MOUTH.

THE Mucus of the mouth serves a variety of purposes. The power it possesses of protecting the surface which it shields, from mechanical injuries, has already been mentioned. It has an equal power of protecting the part covered by it, from chemical injurious impressions. It prepares the substances that are brought in contact with it for absorbtion. It absorbs water, and thus keeps the parts damp and soft and pliable, and in a condition to allow a free osmotic circulation through it. It also serves as a safety valve or outlet for various morbific materials that may have accumulated in, or have been generated within the fluids of the mouth.

The power of the mucus to protect the mucous membrane from mechanical and chemical injuries may be observed at or near all the outlets of the body. In inflammation of the nose, where the nostrils lose the power of secreting mucus, the nasal passages are acted upon by the air inhaled in the inspirations and smarting, and a burning sensation follows. The usual forms of pain in the earlier stages of what is called a "cold in the head" are illustrations of the suffering that follows the absence of mucus.

When a person has "taken a cold" and the ordinary cutaneous transpiration has been checked, an increased flow of mucus may serve as a drain for the morbific materials. Diarrhœa often serves to furnish an outlet for deleterious agents that have found lodgment in the blood. These facts were perceived early, and hence these agents, as the mercurials, that cause a greatly increased flow of saliva and mucus in the mouth, have been considered specially valuable for the purpose of depurating the system of morbid materials not easily gotten rid of by other means.

The cyanide of potassium in the mucus of the mouth, as is not to be found in any other part of the system, doubtless subserves some useful purpose, but what purpose has not been determined.

The *Water* of the mouth is secreted in part, by what are called the *arborescent* glands. These appear as simply branched hollowers in the mucous membrane. They may be compared to a bunch of grapes, or to places from which a bunch of grapes had been dissolved and removed. In these glands the papillæ cease at the outlet of the hollow or tube, which is enclosed with a net-work of blood vessels and lined with epithelium. The drops of water that are poured out on the lips come from the arborescent glands of these parts; others are situated in the cheek and on the tongue. The salivary glands are only arborescent glands of a large size, and seated deeper in the tissues than those of the lips, cheeks or tongue.

The sub-lingual, the sub-maxillary, and the parotid glands all belong to the arborescent division, and all secrete a transparent fluid which is very near pure water, and the amount of their excretion in the aggregate is quite large.

In addition to the fluids poured into the mouth by the follicular glands,—the mucus; and the fluids secreted by the arborescent glands—the water; the mouth is supplied with moisture which exudes directly through the walls of the vessels that abound in the mucous membrane just under the epithelium. This transudes in the same way as the fluids of the lungs is transuded which keeps those organs moist.

Much fluid is excreted into the mouth by the various means indicated, compared with small amount of those fluids which are absorbed again from the mouth. But there are certain articles of the more rare and volatile character that seem to be readily absorbed from the mouth. Among these may be named alcohol, tobocco, onion, and other fancy articles that are taken into the mouth. All the various fluids of the mouth, *combined* from what is termed the *saliva.*

The *Pathological* conditions of the mouth that are directly connected with the fluids of the cavity, may be stated to be :

Arrested Secretion,
Acute Catarrh of the Mouth,
Chronic Catarrh of the Mouth,
Excessive Formation of Epithelium,
Acute Inflammation,
Anæmia.

These various conditions may be present, either one alone and unaccompanied with any one of the others, or they may occur two or more of them at the same time; or one condition may obtain in one part of the buccal cavity while another condition is observed in another part of the mouth.

The secretions may be arrested either from a defective supply of blood to the secretory organ, or from defective innervation of the organ, or from an organic disease of the organ or some one or more of its structures which diminishes its secretory power.

In many forms of fever, where the blood is changed by a poison absorbed into it or formed within it, or in inflammation where the part inflamed yields a poison to the blood and thus poisons that fluid, the tongue may have a darker color than natural, with a sticky and opaque epithelium, and dry, or but imperfectly supplied with moisture, and if the mouth is left open but for a short time, and the saliva is not carefully placed upon the tongue quite frequently, the breath as it passes, dries up the moisture leaving the tongue quite dry and liable to fissures.

As the surface of the tongue becomes dried, the walls of the sub-epithelial capillaries crack, the blood, or at least the serious portion of it oozes out, and drying upon its surface, gives to the tongue a brown or reddish-brown coat.

The diminished secretion, at times, where the blood has been poisoned as here indicated by fever or inflammation, allows the fluid exuded from the fissured capillaries to accumulate in quite large quantities, and if there is much of the red portion of the blood in it, it is tough; and when removed, comes off in flakes, leaving the surface of the tongue red.

This redness and rawness of the tongue indicates that the fluids of the system are seriously poisoned, and the vitality of the mucous membrane so low that there is danger of its sloughing and forming ulcers quite difficult to heal. Especially will there be great danger indicated, if the patient has taken mercurials or antimonials or any other medicines tending to depress the vitality of the organism.

The *Treatment* for arrested secretion of the fluids of the mouth, when caused by a poisoned condition of the blood must be governed by the knowledge that this poisoned state of the fluids seldom continues long, for as the disease runs its course unembarrassed, it tends to a speedy termination, as small pox and measles tend to a speedy termination.

While the arrest of the secretion lasts, but very little of the ordinary foods can be absorbed. Amylaceous foods, which require to be acted on by the saliva, not meeting with the required fluid, are hence not prepared for absorption and nourishment of the patient. But albuminous foods, where the albumen is properly suspended in a sufficiently large quantity of water that the water may take the place in the stomach of the fluids, usually furnished that viscus by the mouth, can be absorbed into the system and serve as nutrition. Hence beef-tea, broth, soup, and other solutions of animal food are far the best in those conditions of the system where the tongue is dry and dark and fissured. A little gelatinous matter, or even a solution of gum or mucilage, taken in the mouth may serve to shield and protect the dry and exposed mucous surfaces, but such solutions retard absorption and should never be taken in more than quite small quantities at a time. Whatever animal food is given, it should be suspended in plenty of water when there are indications from the tongue that the esophagus or stomach are lined with a superabundance of epithelial scales, for those epithelial scales will absorb a considerable portion of the water. But if the stomach is raw and has a surface unprotected by the epithelium, the food should contain less water and be boiled until it is almost or quite a jelly.

As this condition of arrested secretion is usually but of short duration and tends to recovery, proper care in regard to food is of more importance than in regard to the special medicines used.

Stimulants of a permanent character, and especially those that act specifically on the mucous membrane, will aid to restore the arrested secretions of the mouth. The tincture of xanthoxylum berries appears to be specially valuable. The alcohol has a tendency to prevent destructive change in the tissues, while it and the prickly ash both rouse the nervous energy, and the glandular activity of the parts. Sugar also has a preservative power, and as it is changed to lactic acid in the stomach it aids digestion, when given in proper quantities and at proper intervals.

Malt liquors, wine and brandy are of late years being considered almost indispensable in this condition of the system. Tonics, as chamomile flowers, hydrastis, canadensis root, gentian, quassia and other bitters have a direct tendency to arrest the degenerative changes of the tissues, and thus the ravage of the diseases.

Proper care must be taken to keep the emunctories of the system open by apperients, injections, diaphoretics and diuretics, and free bathing and friction of the skin.

